# Desafíos y aprendizajes para la promoción de la salud durante la pandemia de la COVID-19 en Chile. Un análisis de experiencias locales desde la salud colectiva

**DOI:** 10.1177/1757975920986700

**Published:** 2021-06

**Authors:** Maria Sol Anigstein, Soledad Burgos, Sebastián Medina Gay, Karen Pesse-Sorensen, Pamela Espinoza, Carolina Toledo

**Affiliations:** 1 Escuela de Salud Pública Salvador Allende G, Facultad de Medicina Universidad de Chile, Santiago, Chile; 2 Departamento de Antropología, Facultad de Ciencias Sociales, Universidad de Chile, Ñuñoa, Santiago, Chile; 3 Facultad de Medicina, Pontificia Universidad Católica del Ecuador, Quito, Ecuador

**Keywords:** promoción de la salud, acción comunitaria, determinantes de la salud, equidad / justicia social, Latinoamérica, empoderamiento / poder

## Abstract

La Promoción de la Salud (PS) es una función esencial de la salud pública que se ha puesto en tensión frente a la pandemia de la COVID-19, dado que los discursos y estrategias basados en la prevención y curación de la enfermedad han invisibilizado las condiciones de vida e inequidad que son centrales para la PS. La salud colectiva latinoamericana plantea cuestionamientos prácticos y epistemológicos sobre las acciones ante la epidemia en los países del Sur Global, proponiendo enfoques alternativos al paradigma biomédico y a lo que este entiende como PS. Desde la salud colectiva, la PS tiene como elementos centrales la autonomía de las comunidades, la importancia de sus saberes, y el fomento de acciones colectivas territoriales. Este artículo, a través de una cronología crítica de la pandemia en dos territorios, describe situaciones documentadas que ponen en evidencia el despliegue de condiciones esenciales de posibilidad para una PS desde la salud colectiva, es decir, el papel de la justicia social en la organización comunitaria, la perspectiva territorial y los procesos emancipatorios y de construcción de autonomía. Los casos analizados corresponden a un territorio insular del sur de Chile y a una comuna urbana de la capital del país, elaborados mediante el involucramiento directo de los investigadores y una revisión documental y de prensa. Sus antecedentes y contextualización evidencian las modalidades concretas que toma la PS durante la pandemia en dos contextos con características diversas, permitiendo identificar desafíos y arribar a aprendizajes iniciales sobre el desarrollo de una PS territorialmente situada.

## Introducción

Para la salud pública hegemónica, la PS está basada predominantemente en la educación para la salud desde un enfoque biomédico, unidireccional e individual, orientado a cambiar “estilos de vida” considerados nocivos (1). En la pandemia de la COVID-19 esto se traduce en mensajes que enfatizan la responsabilidad individual para adoptar medidas de protección, sin considerar la inequidad estructural de nuestros países del Sur Global. En consecuencia, las medidas preventivas resultan imposibles de cumplir o traen serias consecuencias en la vida de grandes segmentos de la población, además de descrédito y desconfianza respecto de la institucionalidad política y las autoridades sanitarias.

Una perspectiva alternativa de la PS es la propuesta por la salud colectiva, la cual se funda en los principios de esta vertiente crítica de la salud pública en América Latina (2–5) que cuestiona la orientación positivista, funcionalista y reduccionista del enfoque hegemónico. La salud colectiva estudia los fenómenos de salud-enfermedad-atención desde una perspectiva histórica y política, como procesos dialécticos construidos y producidos colectivamente, cuyos elementos centrales son: reproducción social, producción económica, clase social, cultura, etnia y género (3,6).

Desde la salud colectiva, la emancipación de los colectivos (7) en relación con sus territorios es el motor central para transformar las condiciones de vida y de salud (8). Dicha emancipación incluye la construcción de autonomía en la producción y uso de los conocimientos (9), así como una justicia epistémica, esto es, la posibilidad de construir y validar la diversidad de narrativas acerca de la realidad y no sólo las aceptadas por la ciencia moderna (10).

Este artículo propone una reflexión crítica sobre las condiciones de posibilidad de la PS desde la salud colectiva en pandemia, basada en la descripción cronológica de la respuesta institucional y comunitaria ante la COVID-19 en dos territorios de Chile.

## Materiales y métodos

Este artículo expone la primera etapa de un estudio mayor acerca de la PS en pandemia desde la salud colectiva, que corresponde a un estudio de caso múltiple con enfoque holístico y metodología cualitativa (11). Los casos fueron seleccionados respondiendo a un criterio de conveniencia: territorios en los cuales los/as investigadores/as han desarrollado trabajo académico y de activismo antes y durante la pandemia. Considerando la diversidad territorial de Chile, se decidió seleccionar un territorio urbano y uno rural, para generar contrapuntos en la reflexión, considerando que las problemáticas, los actores y las acciones son también diversas y responden a las condiciones estructurales y de la historia de cada territorio. Se suma como criterio de selección, el ser contextos de conflictividad social y ecológica que durante la pandemia se agudizan de forma diferencial en cada territorio: en el caso de Chiloé, insularidad y extractivismo, y en el caso de la Granja, pobreza y marginación.

Como elemento base para la reflexión, se confeccionó una cronología de la pandemia en los dos territorios a partir de una metodología común: 1) revisión bibliográfica acerca de la historia, demografía y características sociosanitarias locales, sobre la base de la información pública más actualizada en los sitios webs oficiales ministeriales y de los municipios (censo, datos del Ministerio de Salud, vivienda y reportes estadísticos por comuna, entre otros); 2) revisión de artículos en prensa local y nacional, publicados en la web privilegiando medios de comunicación ‘alternativos’ y prensa local, dada la escasa visibilización de este tipo de acciones comunitarias en los medios de comunicación masivos; y 3) revisión de redes sociales, como Facebook, Instagram, Twitter y YouTube, que abordaban acciones sociosanitarias locales, antes y durante la pandemia, comunicaciones creadas en su mayoría por las propias organizaciones de base. Tanto para artículos de prensa local como para redes sociales, se emplearon los términos pandemia, la COVID-19 y afines; Chile, La Granja, Chiloé, con el fin de localizar notas y comunicados atingentes al período.

Esta cronología no pretende linealidad sino relevar el espacio-tiempo social, dinámico, con múltiples recorridos y momentos críticos, lo cual permite un análisis comprensivo del contexto de vida de las personas (12). Para su confección, los puntos temporales claves abarcaron desde el 18 de octubre del 2019 hasta junio del 2020.

## Resultados

### Breve contextualización del Chile que despertó

Chile actualmente cuenta con una población de 19,5 millones de habitantes quienes, como consecuencia de la profundización del modelo neoliberal, experimentan graves y diversas desigualdades sociales, y ocupa el tercer lugar dentro de los países de la OECD con mayor inequidad (índice Gini de 0,47) y con menor gasto en protección social (11,2% del PIB) (13). Desde octubre del 2019 ocurren masivas manifestaciones en el llamado “estallido social” por demandas sociales acumuladas desde la dictadura de 1973-90, lo que configura un escenario clave para una posible transformación social (14). Este movimiento se extendió con diversas intensidades en los diferentes territorios del país hasta marzo del 2020, momento en el que se presentaron los primeros casos de la COVID-19. La Granja es uno de los territorios donde las organizaciones sociales se desplegaron con gran intensidad en la revuelta social; misma que llegó también al territorio insular de Chiloé, pero con otras dinámicas y expresiones.

### La Granja: urbanización popular

La Granja es una comuna (municipio) del sector suroriental de la ciudad de Santiago, con una población de 116.571 habitantes. Presenta alta densidad poblacional (11.657,1 habitantes/km2) en rápido aumento por migración (15). En múltiples indicadores la comuna supera los promedios nacionales, como en el índice de pobreza multidimensional, hacinamiento, y carencia de servicios básicos (16). Está emplazada en el denominado “Cordón Industrial Santa Ana”, compuesto por una minera y una curtiembre, entre otras industrias, lo que representa un foco de contaminación ambiental y riesgos para la salud que han sido denunciados en reiteradas ocasiones por los vecinos (17).

En La Granja la inequidad es visible en la segregación territorial y en un ordenamiento, infraestructura y servicios que responden a lógicas de mercado. Su poblamiento comenzó en los años 1960 con viviendas autoconstruidas sobre terrenos rurales, con apoyo de la Corporación de Vivienda o mediante “tomas” y campamentos en terrenos que habían sido entregados a las familias en la “Operación Sitio” con participación de los comités de “Los Sin Casa”. Inicialmente estas viviendas no contaban con servicios básicos como: agua potable y alumbrado público; estos se obtuvieron gracias al trabajo de sus pobladores. Con el Golpe de Estado de 1973, estas poblaciones dejaron de recibir ayuda y las condiciones de vida se precarizaron (18). Durante el “estallido social” del 2019 se constituyó la Asamblea Metro La Granja, instancia de organización social local desde la cual se han realizado varias acciones vinculadas principalmente a las detenciones de manifestantes (19).

### Chiloé: insularidad y extractivismos

El archipiélago de Chiloé está ubicado en la X Región de Los Lagos, en el sur de Chile. Tiene 168.185 habitantes con casi un tercio residente en zonas rurales y 34,8% que declara pertenecer al pueblo mapuche-huilliche (15). Es un territorio atravesado por tensiones sociohistóricas y políticas, principalmente en relación con el Estado centralista, el extractivismo y las migraciones desde y hacia el continente. Entre los extractivismos (forestal, minero, energético y acuícola, entre otros), la acuicultura intensiva del salmón ha estado involucrada en los procesos más críticos de transformación socioambiental de los últimos 40 años, con impactos ecológicos derivados de la sobreexplotación, mal manejo de los recursos marinos, desechos y accidentes^1^ (20). En relación con estas transformaciones han emergido diversas organizaciones sociales por la defensa del territorio, de base comunitaria, ecologistas e indígenas. Estas organizaciones se abocan a la defensa del agua (21) y se movilizan por daños o amenazas inminentes al medio ambiente y a la soberanía insular, como las movilizaciones del “mayo Chilote” en el año 2016 frente a la masiva mortandad de especies marinas (22,23). Durante el “estallido social” del 2019 se conformaron asambleas ciudadanas en las principales ciudades del archipiélago (Ancud, Castro, Chonchi, Quellón) que retoman, además del rechazo al gobierno y a la clase político- partidaria nacional, estas temáticas locales (24).

### Cronología de una epidemia

En Chile, el primer caso de la COVID-19 se hizo público el día 3 de marzo (25), dando lugar a una serie de reacciones en las que se percibe una respuesta pasiva y errática por parte del gobierno. Inicialmente se intentaron acallar las voces que daban importancia al brote epidémico (26). Pocos días más tarde (el 18 de marzo), siendo innegable el arribo de la pandemia y con 238 casos diagnosticados, se decretó un “estado de excepción constitucional de catástrofe” en todo el país (27). Esto incorporó a “las fuerzas armadas en el control de la pandemia” con el fin de asegurar, entre otros puntos, la “restricción de reuniones en espacios públicos”. Simultáneamente se intentó dar un mensaje de tranquilidad a la ciudadanía, asegurando que el país “está preparado”. Luego se añadió un “toque de queda” nacional desde las 22:00 hasta las 5:00 horas (28). Estas decisiones y acciones carecieron de participación de la comunidad y de los equipos de atención primaria, excluidos de la estrategia de contención de la epidemia, lo que implicó que fueran ampliamente cuestionadas por la población, la cual las asoció con la represión y el control de las movilizaciones durante el estallido social (29).

La siguiente estrategia del gobierno consistió en establecer cuarentenas dinámicas; al inicio en las comunas con mayor número de casos correspondientes a sectores socioeconómicos altos y, a medida que aumentaban los casos, en los sectores más vulnerables del Gran Santiago. Sin embargo, los casos continuaron aumentando exponencialmente, develando la ineficacia de esta medida, por lo que el 13 de mayo se decretó una cuarentena total en toda la Región Metropolitana. En esta etapa se verificó un acelerado aumento en las tasas de incidencia de la COVID-19 en las comunas más vulnerables, mientras que las inicialmente afectadas, lograban controlar mejor el aumento de casos. Esta situación se asoció con factores que impiden a la población cumplir con la cuarentena: hacinamiento, pobreza y alto porcentaje de empleos informales, entre otros (30,31).

Las deficiencias o ausencias de medidas de seguridad social para responder a algunas de las consecuencias de la enfermedad y de la crisis económica generada por esta, llevaron a nuevas movilizaciones sociales en varios sectores de Santiago. Las primeras “protestas por el hambre”, en la comuna de El Bosque en mayo, marcaron un importante hito (32). Al mismo tiempo, surgieron respuestas solidarias enfocadas en la sobrevivencia, entre las que se destaca la organización de ollas comunes. Estas son iniciativas colaborativas entre vecinos de las poblaciones más vulnerables y afectadas por la crisis (33). El gobierno por su parte respondió con el anuncio de entrega de cajas de mercadería (alimentos) justamente a estos grupos poblacionales (34).

En La Granja, al igual que en otros territorios urbanos, las reacciones comunitarias ante la pandemia comenzaron durante los primeros días de marzo. En contexto de alerta, la Asamblea Territorial Metro La Granja constituyó un Comité de Emergencia (CE) de la Asamblea, que posteriormente se articuló con el Movimiento Salud en Resistencia (MSR). El MSR surgió en Santiago durante el estallido social de octubre 2019 como una brigada integrada por estudiantes y trabajadores del área de la salud que otorgaba primeros auxilios, atención médica, resguardo y apoyo a heridos durante las manifestaciones. Ante la pandemia sus actividades se volcaron a apoyar acciones sanitarias autogestionadas por distintas asambleas populares en sus territorios, como una estrategia de contención comunitaria y respuesta a la política gubernamental. (35) En este contexto, se realizó un primer operativo del MSR en La Granja, en conjunto con el CE local (26 de abril de 2020). Sus acciones iniciales fueron: un “catastro epidemiológico popular” realizado por los mismos vecinos, desinfecciones de casas y ollas comunes realizadas por jóvenes que hacían parte de la denominada “primera línea” durante el estallido social, educación preventiva en salud y articulación con personal de salud. Así se comenzó a desarrollar un complejo plan territorial frente a la COVID-19 bajo el lema “solo el pueblo ayuda al pueblo” (36,37).

A través de sus redes sociales, el CE ha expresado la importancia de enfrentar en forma solidaria lo que llaman la “cuarentena de hambre” impuesta por el gobierno. En redes sociales publican que no es posible afrontar esta cuarentena “sin pensiones, sin plata, sin posibilidad de abastecerse”. Hacen un llamado a la autoorganización y articulación con otras organizaciones sociales para levantar Comités de Emergencia en nuevos territorios, y resolver en forma colectiva las necesidades de salud de la población que, desde su perspectiva, ha sido abandonada por el sistema de salud público y por los municipios (38).

En Chiloé la epidemia se hizo visible en los medios locales hacia fines de marzo, con el primer caso provincial de la COVID-19 (39). La reacción inicial ante el miedo al contagio fue que las comunidades se movilizaron para exigir, a través de bloqueo de carreteras y rampas, primero el cierre y luego la instalación de un “cordón sanitario” como medida estricta de control del acceso al archipiélago. En particular, se solicitaba control en el cruce sobre el Canal de Chacao, que une la isla grande con el continente chileno a través de barcos ferry de alta frecuencia y capacidad de carga, y en otras vías de acceso a las ciudades principales y a algunas empresas (40). Si bien esta acción fue percibida como necesaria, poco tiempo después se hizo inefectiva ya que se observaba gran circulación de vehículos con trabajadores pese a la barrera sanitaria establecida por las autoridades, situación que fue denunciada como una irregularidad en el manejo de epidemia la pandemia (41). En particular, se describe el tránsito de camiones salmoneros, miticultores, forestales y que sacan “pompón” *(Sphagnum*, musgo vital para la homeostasis hídrica del suelo), lo que corresponde a las principales actividades extractivistas del territorio (42).

De este modo, las primeras tensiones se presentaron en la relación con el empresariado salmonero, mitilicultor y transportista, y sus propuestas de acciones mitigadoras para continuar con el flujo de camiones extractivistas. Algunas de las medidas implementadas plantearon dudas sobre sus posibles efectos adversos para la salud; por ejemplo, los “túneles sanitarios” utilizados para rociar vehículos y personas, los que tuvieron que ser suspendidos por la autoridad sanitaria ante los reclamos comunitarios (43). Asimismo, se evidenciaron respuestas represivas policiales que fueron informadas en declaraciones a los medios que exponen las condiciones de vida precarias para hacer frente a la pandemia (44).

Ya avanzada la pandemia, se abrieron espacios de reflexión en los círculos comunitarios (45,46). Uno de los más relevantes, corresponde a la iniciativa de la Escuela Superior Campesina que organiza el Quelcún constituyente que, en reacción a la pandemia y la invisibilización de las organizaciones sociales (47), ha creado espacios de formación ciudadana abordando las problemáticas socioambientales más críticas en el territorio.

## Reflexión y conclusiones

Siguiendo las tres condiciones esenciales de la PS desde la perspectiva de la salud colectiva, a saber: justicia social, territorio y autonomía-emancipación, es posible abordar ciertas reflexiones a modo de conclusiones.

Una primera condición es la *justicia social* como motor de las acciones comunitarias y telón de fondo que parece atravesar toda la realidad nacional, adoptando diversas formas. En algunos casos se trata de un aspecto latente y fundido con reivindicaciones territoriales y culturales que, junto con demandas sociales relativas al trabajo, también compromete demandas por justicia ambiental (como en Chiloé). En otros (como en La Granja), se reinventa para constituir un discurso en torno a la desigualdad social y la violencia de Estado (48) en clave pandemia.

Este trasfondo en que la justicia social opera como movilizador, haría parte de lo que De Souza Porto (49) ha elaborado como “crisis de las utopías sociales emancipatorias por la justicia” que tienen expresión en diferentes escalas (local, nacional, regional, planetaria) y dimensiones de justicia: social, sanitaria, ecológica y cognitiva.

La segunda condición es la perspectiva *territorial* tanto en la problematización y toma de conciencia de las condiciones estructurales e injusticias, como en las estrategias y acciones que se llevan a cabo por parte de la comunidad. Dicha dimensión permite comprender cómo el modelo de ciudad neoliberal configura, en la comuna de La Granja, espacios urbanos de sacrificio social que poseen un efecto performativo sobre los territorios y sobre los cuerpos (50). Dichos espacios se caracterizan por expresiones de degradación de las zonas urbanas, tales como empobrecimiento, hacinamiento, condiciones precarias de vivienda, dificultad de acceso a servicios y marginalidad, componentes influyentes en la determinación social de los procesos de salud y enfermedad (50). En el caso de Chiloé, el modelo de desarrollo extractivista y la condición de insularidad configura este territorio como un enclave extractivista que depende de “redes de conectores”: vías de transporte libre para la salida y entrada de recursos naturales, personal, insumos, maquinaria, energía, etc. (51).

La manera en la que el modelo de desarrollo y económico configura los territorios en Chile se vincula a la vez con los tipos de respuesta que parecen estar emergiendo en cada territorio. En el espacio urbano, la organización y las acciones prestan servicios que debieran ser proporcionados por el Estado, por medio de catastros, seguimientos, operativos de salud, desinfecciones y ollas comunes, lo cual es facilitado por la disposición urbana y la cercanía. En Chiloé la estrategia inicial es cortar caminos y crear un cordón sanitario comunitario, lo que, al transformarse en medida sanitaria estatal, se negocia con los empresarios y se permite la reanudación de la circulación de transporte.

La tercera condición esencial de posibilidad de la PS es que exista un despliegue de ‘procesos emancipatorios y de construcción de autonomía’. En los casos analizados hay dos elementos que parecen ser fundamentales. En primer lugar, la estrategia de afrontamiento de la pandemia por el gobierno, que plantea la retórica de “estamos preparados” sin disponer de acciones concretas de protección social. Se intenta continuar con la actividad económica, pero con un aumento de las medidas represivas, lo cual refuerza y abre el espacio para una organización comunitaria autogestionada dado el vacío estatal en la contención de la pandemia y la falta de apoyo a las comunidades. Esto es visible en los operativos de salud realizados por el MSR en La Granja, así como en los cordones sanitarios y los cortes de ruta comunitarios en Chiloé.

En segundo lugar, la trama recompuesta de la organización social y comunitaria durante el estallido social, la baja credibilidad y pérdida de legitimidad de las autoridades, así como la represión estatal sufrida por parte importante de la población, provocan que las medidas sanitarias establecidas por el gobierno central no resulten confiables y sean leídas como un intento por privilegiar la salud de la economía antes que la salud de la población. En tal sentido, la desconfianza y baja legitimidad, así como la desconexión de las autoridades, en sinergia con la ausencia del Estado y la exclusión de parte importante de la población, han implicado la construcción de otro mundo “paralelo”, en lo que puede reconocerse una actitud emancipadora (7), en busca de mejorar las condiciones de vida (8). En Chiloé este parecía estar enfocado en prevenir la llegada de la enfermedad, las tensiones generadas por el no respeto de los cordones sanitarios activaron una red de organizaciones con demandas más amplias y anteriores a la pandemia.

Retomando los elementos que definimos como centrales para una PS desde la perspectiva de la salud colectiva, es posible sostener que en el contexto de la pandemia se han desplegado formas de organización colectiva arraigadas en procesos históricos de mayor alcance y vinculadas al estallido social en La Granja, y a demandas medioambientales e indígenas en Chiloé. Estas acciones han estado ancladas y responden a las características de cada uno de los territorios y, a la vez, han estado orientadas a la justicia social por un lado y a la posibilidad de construir autonomía por el otro. Es decir, parecieran estar en curso formas locales de PS, basadas en estos tres principios, búsqueda de justicia social, desde una perspectiva territorial, por medio de acciones autónomas que son parte de procesos emancipatorios de largo aliento, profundizados en el contexto de la pandemia.

Esta forma de comprender la PS presenta grandes desafíos y significa aprendizajes. En Chile, la PS como estrategia de Atención Primaria de Salud, si bien reconoce el enfoque de Determinantes Sociales en sus lineamientos, lo cual puede vincularse a la búsqueda de justicia en todos sus planos, ha sido cuestionada tanto por sus modos de comprensión como por su praxis. Esto se debe a que está sobrecentrada en la biomedicina y en enfoques preventivos (52) que se apoyan en discursos normativos relativos a estilos de vida saludables (53), muchas veces ajenos o descontextualizados de la realidad de los territorios, abriendo una brecha conceptual y operativa que se aleja de la perspectiva de salud colectiva como vertiente analítica. En el transcurso de la pandemia, no solo se visualiza la ausencia de una estrategia nacional y local a nivel territorial en todas las dimensiones del cuidado de la salud, sino un redireccionamiento de los recursos humanos, incluidos los de la PS, a tareas preventivas únicamente frente a la COVID-19, lo cual refuerza la ausencia y la necesidad de autogestión en salud de la propia comunidad. Consistente con ello, la participación social–otro eje central en las estrategias de PS declaradas en Chile (54)–no hace parte de instancias de decisión local, lo cual si bien puede entenderse como una deriva de la desarticulación funcional de los equipos de salud locales en la emergencia de la pandemia, también sugiere una interrelación empobrecida de los equipos de salud con la comunidad, ya previo a la pandemia, que restringiría la posibilidad de emprender acciones colectivas y mancomunadas. Esto pone en evidencia que, tanto para la política pública y la academia, como para los equipos de atención primaria en cada territorio, es necesario repensar la estrategia de PS desde un reposicionamiento que permita acompañar y potenciar este tipo de acciones ya desplegadas por las propias comunidades.

## Notes

1. De alto volumen e intensidad, el extractivismo salmonero en la Región cosechó 923.900 toneladas en el año 2018 (55) y reportó ingresos de 1,78 mil millones de dólares (56).
